# An integrated bioinformatic analysis of microarray datasets to identify biomarkers and miRNA-based regulatory networks in leishmaniasis

**DOI:** 10.1038/s41598-024-63462-5

**Published:** 2024-06-05

**Authors:** Amir Savardashtaki, Shayan Khalili Alashti, Asma Vafadar, Mahboubeh Sadeghi, Maryam Baneshi, Kimia Sadat Hashemi, Jafar Karami, Antonio Muro, Raúl Manzano-Roman, Sajad Rashidi

**Affiliations:** 1 Department of Medical Biotechnology, School of Advanced Medical Sciences and Technologies, Shiraz University of Medical Sciences, Shiraz, Iran; 2 Infertility Research Center, Shiraz University of Medical Sciences, Shiraz, Iran; 3 Epilepsy Research Center, Shiraz University of Medical Sciences, Shiraz, Iran; 4 Student Research Committee, Shiraz University of Medical Sciences, Shiraz, Iran; 5 Department of Medical Genetics, Medical University of Innsbruck, Innsbruck, Austria; 6 Molecular and Medicine Research Center, Khomein University of Medical Sciences, Khomein, Iran; 7 Department of Medical Laboratory Sciences, Khomein University of Medical Sciences, Khomein, Iran; 8 Infectious and Tropical Diseases Group (E-INTRO), Faculty of Pharmacy, Institute of Biomedical Research of Salamanca-Research Center for Tropical Diseases at the University of Salamanca (IBSAL-CIETUS), University of Salamanca, 37008 Salamanca, Spain

**Keywords:** Leishmaniasis, Biomarkers, Regulatory network, Gene expression profiling, miRNA, Microarray, Biotechnology, Cell biology, Immunology, Microbiology, Molecular biology, Diseases, Molecular medicine, Pathogenesis

## Abstract

Micro RNAs (miRNAs, miRs) and relevant networks might exert crucial functions during differential host cell infection by the different *Leishmania* species. Thus, a bioinformatic analysis of microarray datasets was developed to identify pivotal shared biomarkers and miRNA-based regulatory networks for Leishmaniasis. A transcriptomic analysis by employing a comprehensive set of gene expression profiling microarrays was conducted to identify the key genes and miRNAs relevant for *Leishmania* spp. infections. Accordingly, the gene expression profiles of healthy human controls were compared with those of individuals infected with *Leishmania mexicana*, *L. major*, *L. donovani*, and *L. braziliensis*. The enrichment analysis for datasets was conducted by utilizing EnrichR database, and Protein–Protein Interaction (PPI) network to identify the hub genes. The prognostic value of hub genes was assessed by using receiver operating characteristic (ROC) curves. Finally, the miRNAs that interact with the hub genes were identified using miRTarBase, miRWalk, TargetScan, and miRNet. Differentially expressed genes were identified between the groups compared in this study. These genes were significantly enriched in inflammatory responses, cytokine-mediated signaling pathways and granulocyte and neutrophil chemotaxis responses. The identification of hub genes of recruited datasets suggested that *TNF, SOCS3, JUN, TNFAIP3*, and *CXCL9* may serve as potential infection biomarkers and could deserve value as prognostic biomarkers for leishmaniasis. Additionally, inferred data from miRWalk revealed a significant degree of interaction of a number of miRNAs (hsa-miR-8085, hsa-miR-4673, hsa-miR-4743-3p, hsa-miR-892c-3p, hsa-miR-4644, hsa-miR-671-5p, hsa-miR-7106-5p, hsa-miR-4267, hsa-miR-5196-5p, and hsa-miR-4252) with the majority of the hub genes, suggesting such miRNAs play a crucial role afterwards parasite infection. The hub genes and hub miRNAs identified in this study could be potentially suggested as therapeutic targets or biomarkers for the management of leishmaniasis.

## Introduction

Leishmaniasis is a widespread tropical and subtropical disease, caused by various species of *Leishmania* spp., which are transmitted through the bite of the female *Phlebotomine* sandflies^1–3^. The estimated epidemiological range of 12 million cases with an addition of 2 million new annual cases, poses a substantial threat of leishmaniasis to public health. It is noteworthy that the reported leishmaniasis cases are concentrated in more than 98 countries across all continents^4^. Leishmaniasis presents with a diverse range of clinical manifestations, encompassing cutaneous leishmaniasis (CL), causing non-lethal skin lesions, and visceral leishmaniasis (VL), commonly called kala-azar, affecting internal organs, leading to pancytopenia, hepatomegaly, and splenomegaly^5^. *Leishmania major, L. tropica, L. aethiopica,* and *L. donovani, L. infantum* have been recognized as the main causative agents of CL and VL*,* respectively^6^.

*Leishmania* protozoan parasites employ different and sophisticated evasion strategies dealing with the host’s immune responses including the inhibition of complement system and modulating anti-parasitic activity of immune T-cells. Furthermore, these intracellular parasites by expressing crucial parasite proteins are able to affect downstream proteins and consequently affect and change the host cell signaling pathways and responses to favor the parasite survival and pathogenesis persistence^7,8^. In this sense, small and long non-coding RNAs are considered as biomolecules involved in many cell regulatory networks (through regulating gene expression) during parasite infections^9,10^. Thus, such pivotal biomolecules can impact key biological processes including immunity and pathogenesis processes of different parasitic diseases such as leishmaniasis^11^.

microRNAs (miRNAs, miRs) are small endogenous ncRNAs implicated in post-transcriptional regulation by binding to the 3’ UTR of target mRNA, leading to mRNA degradation or translational inhibition^12^. They exert a modulatory influence on diverse biological processes including cell proliferation, differentiation, and immune response regulation^13,14^. Consequently, expression and modulation of miRNAs can be implicated in the development of pathogenesis processes of several human diseases including parasitic infections. Therefore, the identification of such biomarkers could provide novel therapeutic and diagnostic prospects for the management of leishmaniasis^15^. Furthermore, unique miRNAs are directly implicated in spike glycoprotein production upon coronavirus (COVID) vaccination which might also open new avenues to promote protective signals during nano-vaccine development in parasitic diseases such as leishmaniasis^16^. There is a great deal of studies that identified various and crucial miRNAs (miRNAs expressed in both parasite and infected-host cells) during leishmaniasis infection^17,18^.

Given the significance of accurate molecular diagnosis in the context of leishmaniasis, this study aims to discover the crucial genes and miRNAs linked to *Leishmania* spp. infection by conducting a meta-analysis of expression microarray datasets using bioinformatic techniques. Additionally, the interaction of the miRNA-hub genes implicated in leishmaniasis along with their associated signaling pathways will also be investigated.

## Materials and methods

### Collection and selection of eligible gene expression datasets

In the discovery step, we identified first microarray datasets that compared gene expression between leishmaniasis patients and healthy controls in different tissues and cell types. Microarray data were retrieved from the Gene Expression Omnibus (GEO, https://www.ncbi.nlm.nih.gov/geo/) database with “*Leishmania”*, “Expression profiling by array”, “microarrays”, and “Homo sapiens” as keywords. During the second step, the selection process for qualified studies and datasets, we followed a number of strict criteria including human case–control studies, gene expression profiling analysis, comparable test conditions, and the downloadable of complete raw and processed microarray data. Other clinical covariates, including age, sex, and therapeutic status were not available for all samples, therefore, to avoid the introduction of false positives by imputation, have not been included. Disqualifying factors for studies included the use of cell lines, exclusive reliance on Real-Time PCR (RT-PCR) profiling, absence of case–control design, and investigation of specific factors' impact on leishmaniasis. The datasets and references that met the aforementioned requirements were all manually reviewed^14,19^.

### Data extraction and processing

For each dataset, the series matrix file was downloaded and processed in several steps including background correction, log2 transformation, and quantile normalization which were performed using R language v4.2.2 and GEO2R analysis software. We used the R packages including Limma, GEOquery, BiocGenerics, Biobase, parallel, reshape, reshape2, ggplot2, grid, plyr, dplyr, data.table, sva, and affy from Bioconductor to process the data. After computation, all datasets related to *Leishmania* infections were merged. During this step, the expression data were mean-centered and reduced to the number of common probes across all data sets^20^. The gene expression data of the merged datasets were batch-adjusted using the ComBat method, implemented in the sva package v3. Assessing the success of batch correction was confirmed by boxplots.

### Differential gene expression screening

For every dataset, analysis to identify differentially expressed genes (DEGs) were developed. Limma package (https://bioconductor.org/packages/release/bioc/html/limma.html), which applies linear models to examine the expression patterns of individual genes, was used to obtain the required tools to analyze DEGs with *T* test. False discovery rate (FDR) was calculated using Benjamini & Hochberg method. The FDR cut-off value for DEGs was below 0.01. By calculating adjusted *p* values and fold changes (FC), we identified genes which exhibited differential expression, defined as those with an adjusted *p* value less than 0.05 and |log FC|> 1.5. Additionally, Heatmap was generated for each dataset using the Python packages (version 3.11) to represent significant DEGs.

### Enriched gene ontology (GO) and pathways analysis

Gene ontology servers as a comprehensive resource for the high-throughput annotation of biological functions. This ontology framework encompasses three fundamental biological dimensions, including the biological process (BP), molecular function (MF), and cellular component (CC). Here, GO enrichment and Kyoto Encyclopedia of Genes and Genomes (KEGG) pathway analyses of DEGs were conducted using the Enrich database (https://maayanlab.cloud/Enrichr/) (www.kegg.jp/kegg/kegg1.html)^21^. To ensure the statistical validity, selection criteria were utilized, which included the presence of at least three genes within a cluster with a GO tree interval ranging from 3 to 8 and a kappa score of 0.4 for pathway network connectivity^22^. Consequently, any *P* value less than 0.05 was considered to possess statistical significance.

### Clustering gene expression data

Python libraries including matplotlib, and seaborn were imported to cluster DEGs and visualize the results. We used hierarchical clustering, a potent technique for examining high throughput expression data. Python determined the degree of similarity among the genes in each set of data, displayed the expression value using colors, and then clustered the genes.

### Protein–protein interaction (PPI) network construction, cluster networks, and identification of hub genes

The Search Tool for the Retrieval of Interacting Genes (STRING) database, available at (http://string-db.org), was utilized to establish a Protein–Protein Interaction (PPI) network. Gene co-expression analysis was subsequently performed on the network, employing a confidence score threshold of more than 0.4 for GSE42088, GSE43661, GSE63931, GSE64610, and GSE69252 datasets and 0.7 for GSE55664 dataset^23^. In order to identify hub genes, a protein–protein interaction (PPI) network was constructed using the software Cytoscape 3.7.1 (http://www.cytoscape.org). Genes that met the following cut-off thresholds were identified as hub-genes: Degree > 10, K core > 2, and max depth > 100. The top 10 hub genes were determined using cytoHubba, employing four algorithms based on closeness, betweenness, and connectivity (degree) methods^14^. The final selection of hub genes was made by finding the intersection between the sets of hub genes obtained from all three algorithms. In addition, the Molecular Complex Detection (MCODE) tool was used to visually examine clusters within the PPI network. The MCODE analysis involved specific parameter settings, including a degree cutoff of 2, a node score cutoff of 0.2, a k-core value of 2, and a maximum depth of 100. Genes exhibiting the highest MCODE scores were identified as potential hub genes. In order to determine the hub genes that were shared among different methods and analysis, a Venn diagram was employed (http://bioinformatics.psb.ugent.be/webtools/Venn/). This Venn diagram facilitated the identification of common hub genes across various approaches.

### Hub gene validation

The predictive effects of hub genes on infection establishment were evaluated by employing receiver operating characteristic (ROC) curve analysis. The diagnostic value of the hub genes was compared by calculating the area under the ROC curve (AUC). The MedCalc version 22 was used to perform this analysis^24^.

### Evaluation of miRNAs-hub genes interaction network

**Figure 1:**
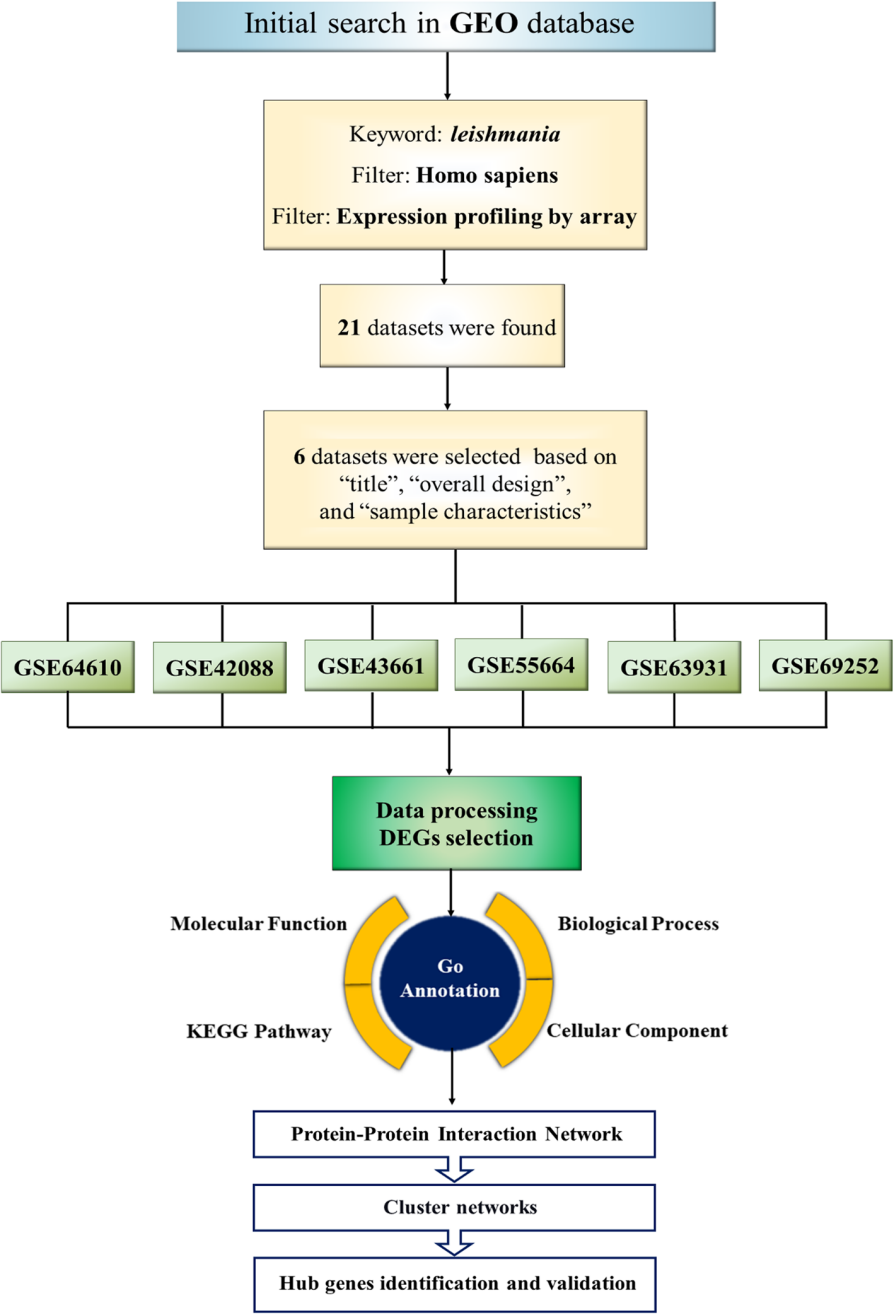
The flowchart of microarray analysis approach towards biomarker discovery in *Leishmania* infections. Six gene expression datasets were downloaded from GEO, and the differentially expressed genes (DEGs) in leishmaniasis patients and healthy controls with an adjusted *P* value < 0.05 and a |log fold change (FC)|> 1.5 were first identified by GEO2R or R language version 4.2.2. Next, Gene Ontology (GO) and Kyoto Encyclopaedia of Genes and Genomes (KEGG) analysis (www.kegg.jp/kegg/kegg1.html) were performed for enrichment analysis of these DEGs. Then, the hub genes were identified by the cytoHubba plugin and the other bioinformatics approaches including protein–protein interaction (PPI) network analysis, and miRNA-hub gene network construction was also performed. This approach promotes a comprehensive understanding of parasite infection and for biomarker discovery useful for early diagnosis.

In order to determine the specific miRNAs associated with the hub genes, we utilized various resources including the miRTarBase database (https://www.mirtarbase.cuhk.edu.cn), miRWalk (https://mirwalk.umm.uni-heidelberg.de/), and TargetScan (https://www.targetscan.org/). In addition to evaluating miRNAs through gene-miRNAs interaction, a comprehensive review of literature studies was also conducted. Furthermore, the miRNet database (https://www.mirnet.ca/) was employed to generate a graphical depiction of the interactions between miRNAs and the hub genes. The steps of microarray analysis approach are outlined in Fig. 1, providing a visual representation of the entire pipeline.

## Results

### Dataset’s characteristics

**Figure 2:**
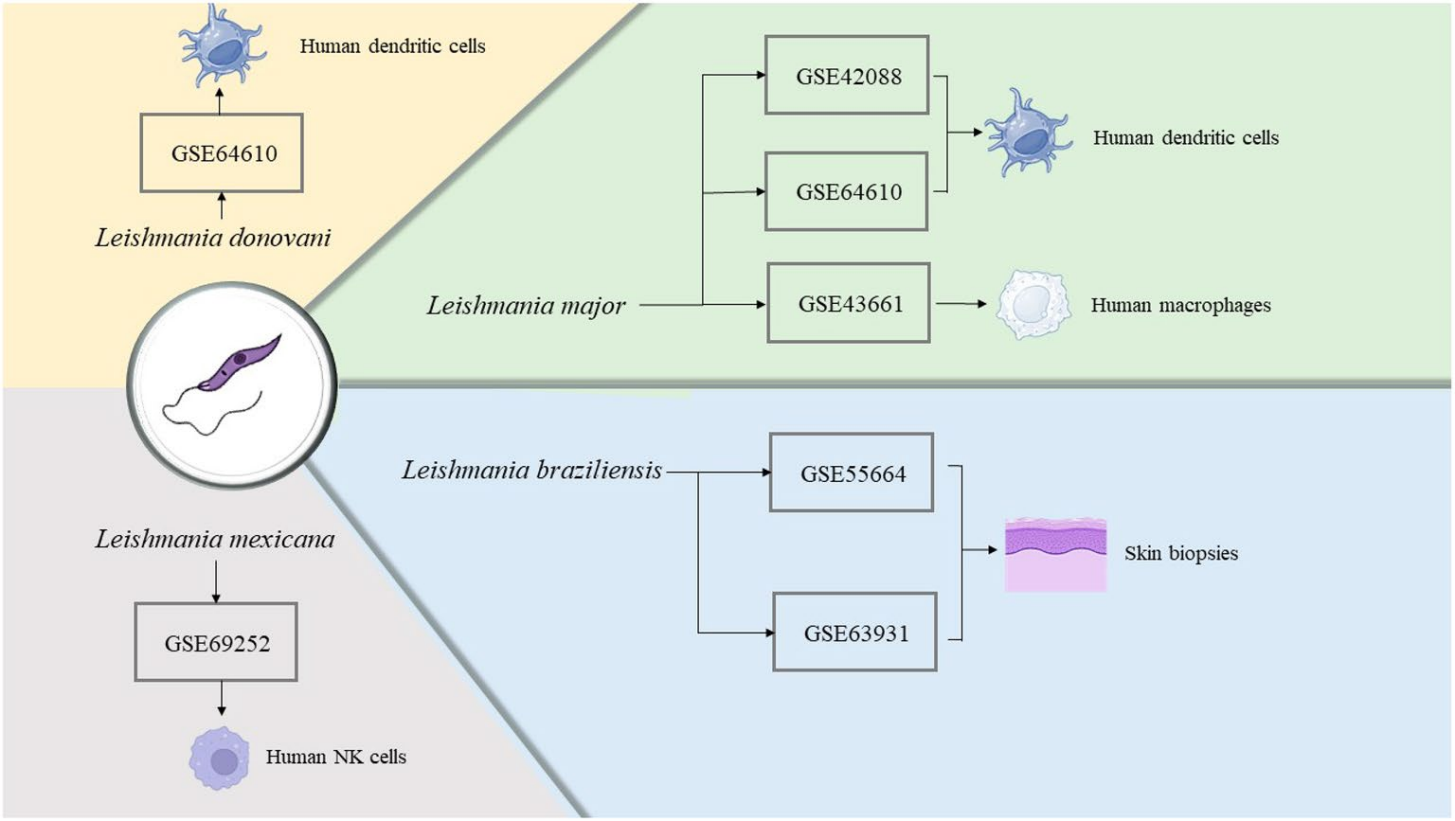
Details of the recruited datasets related to each species of *Leishmania* along with the sort of the studied cells. Datasets belong to different cells of the different species of *Leishmania.*

Our prespecified criteria led to the identification of six datasets, namely GSE69252 and GSE43661 datasets which were based on the GPL6244 platform (Affymetrix Human Gene 1.0 ST Array), while GSE42088 was analyzed using the GPL70 platform (Affymetrix Human Genome U133 Plus 2.0 Array), GSE64610 was based on the GPL16025 platform (NimbleGen Homo Sapiens Expression Array), GSE63931 was evaluated by GPL17077 platform (Agilent-039494 SurePrint G3 Human GE v2 8 × 60K Microarray 039,381), and GSE55664 was related to GPL10558 platform (Illumina HumanHT-12 V4.0 expression beadchip). Table 1 displays the fundamental characteristics of the datasets that were assimilated. Moreover, the microarray analysis employed diverse types of cell sorts which clearly depicted in Fig. 2.

**Table 1:** Distinguishing features of each microarray datasets for the current study.

GEO accession number	Sample (Normal/Leishmaniasis)	Species	Platform
GSE69252	18 (8/10)	*L. mexicana*	GPL6244
GSE43661	27 (15/12)	*L. major*	GPL6244
GSE42088	15 (3/12)	*L. major*	GPL70
GSE64610	20 (5/15)	*L. donovani*	GPL16025
	20 (5/15)	*L. major*	
GSE63931	16 (8/8)	*L. braziliensis*	GPL17077
GSE55664	35 (10/25)	*L. braziliensis*	GPL10558

### Identification of DEGs

The DEGs were attained through the examination of six microarray datasets in correlation with four distinct *Leishmania* species. The results of our mRNA analysis represent a total of 528 DEGs (475 upregulated, 53 downregulated) for GSE55664, 107 DEGs (70 upregulated, 37 downregulated) for GSE42088, 187 DEGs (63 upregulated, 124 downregulated) for GSE43661, 71 DEGs (58 upregulated,13 downregulated) for GSE63931, 118 DEGs (108 upregulated, 10 downregulated) for GSE64610, and 67 DEGs (63 upregulated, 4 downregulated) for GSE69252. Table 2 presents a categorical arrangement of the genes that have been up-regulated and down-regulated, which were detected as remarkably significant in the microarray examination.

**Table 2:** Expressional profiles three top up- and down-regulated DEGs which were ranked by combined Log(FC) and *P* value.

Up-regulated			Down-regulated		
Genes	Log(FC)	*P* value	Genes	Log(FC)	*P* value
GSE55664					
*CXCL9*	6.42	1.31E-23	*PIP*	− 5.8	5.84E−12
*CXCL10*	6.16	2.41E-22	*DCD*	− 5.36	1.22E−07
*IDO1*	5.97	4.32E-19	*SCGB1D2*	− 5.14	1.40E−14
GSE42088					
*INHBA*	4.471384354	0.021640015	*LRRC25*	− 2.792619	0.031868284
*KLK4*	4.286646366	9.49E-05	*GIMAP1*	− 2.603461	2.27E-08
*RGS1*	3.966958814	0.036598559	*GPR82*	− 2.588397	0.043572491
GSE43661					
*CXCL10*	2.350071712	0.012865475	*KRTAP4-6*	− 3.174471	0.000297249
*UBD*	1.848824964	0.018690253	*RNU6-344P*	− 2.723495	0.001334455
*CCL8*	1.53317718	0.020752784	*CDSN*	− 2.598859	0.000517079
GSE63931					
*MMP1*	12.1451723	4.51E-15	*ADH1A*	− 7.771482	7.76E-08
*MMP3*	11.4818036	3.99E-14	*XIST*	− 7.410758	2.85E-04
*CXCL9*	9.2524916	5.78E-11	*FABP7*	− 7.248062	2.00E-06
GSE64610					
*CCRL2*	3.058233	3.17E-09	*ABHD11*	− 1.824708	5.39E-05
*G0S2*	2.936634	9.55E-14	*OTULINL*	− 1.594114	2.24E-07
*MSANTD3*	2.871608	1.51E-08	*SUOX*	− 1.479999	4.43E-03
GSE69252					
*SLC7A11*	4.363233801	4.62E-05	*LGMN*	− 1.840519	0.00430786
*MMP7*	4.038071715	8.52E-05	*MPEG1*	− 1.665258	0.000141278
*LYZ*	3.84952256	7.82E-05	*HLA-DQA1*	− 1.575723	0.001040029

### Gene ontology

**Figure 3:**
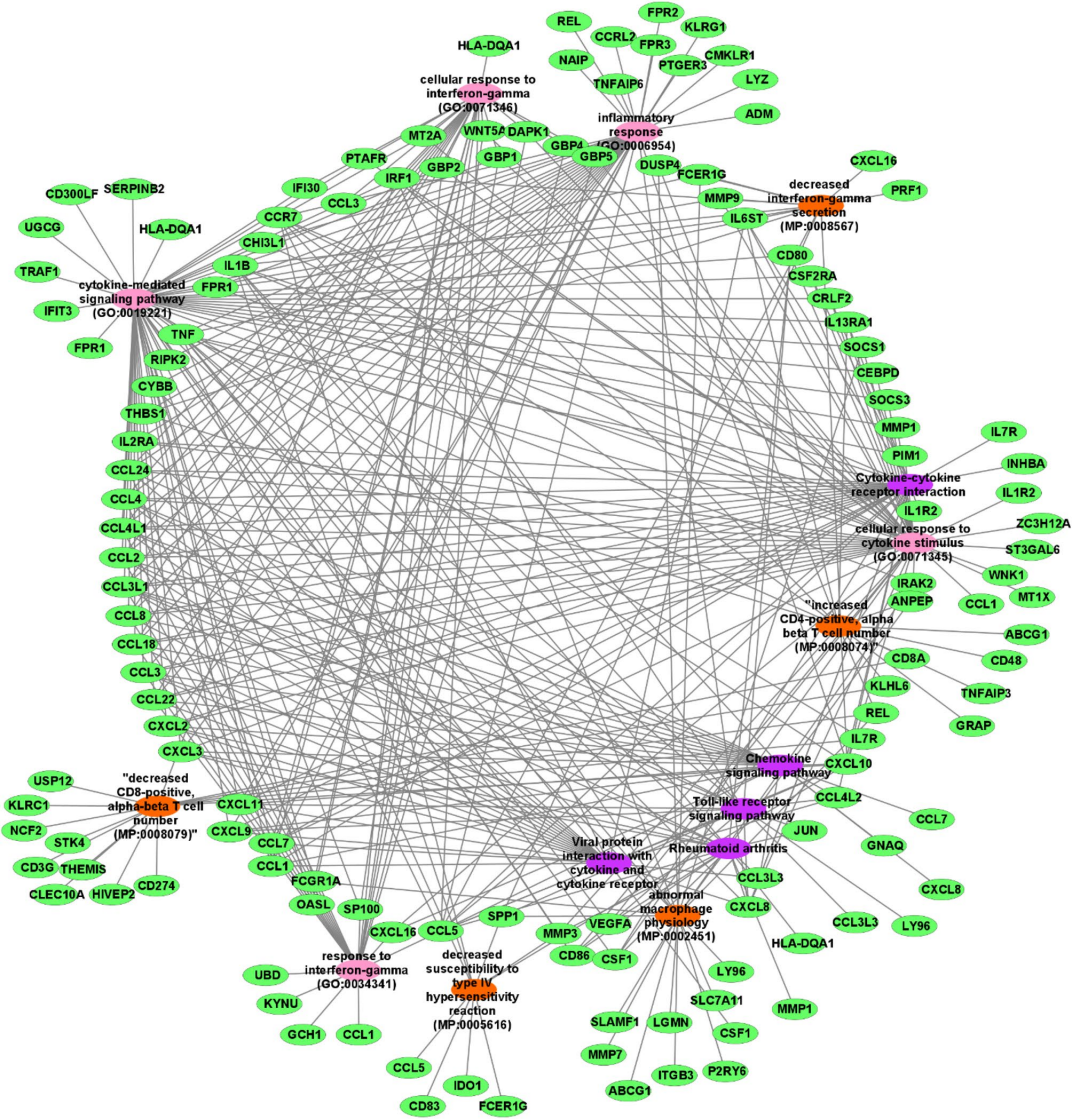
Enrichment analysis associated with DEGs obtained from the Enricher database. Green nodes represent the genes while pink nodes display gene ontology terms, purple nodes show pathways and mammalian phenotype data displayed by orange node.

The current investigation performed a GO enrichment analysis on DEGs to uncover the fundamental biological pathways of leishmaniasis caused by different *Leishmania* spp. The findings of GO clarified that, DEGs are significantly enriched in immunological process, functions and, pathways such as inflammatory response, cytokine-mediated signaling pathway, granulocyte and neutrophil chemotaxis, etc. which is demonstrated in Table 3. The results of enrichment showed that most of the DEGs were involved in cellular response to cytokine stimulus (GO:0071345), cellular response to interferon-gamma (GO:0071346), inflammatory response (GO:0006954), cytokine-mediated signaling pathway (GO:0019221), abnormal macrophage physiology (MP:0002451), decreased CD8-positive, alpha–beta T cell number (MP:0008079), increased CD4-positive, alpha beta T cell number (MP:0008074), and also pathways including rheumatoid arthritis, cytokine–cytokine receptor interaction and, chemokine signaling pathway (Fig. 3).

**Table 3:** Top three of biological processes, molecular functions, cellular components, and KEGG pathways (www.kegg.jp/kegg/kegg1.html).

Datasets	Biological processes	Molecular function	Cellular components	KEGG pathways
GSE69252	Inflammatory response (GO:0006954) Cellular response to cytokine stimulus (GO:0071345) Cytokine-mediated signaling pathway (GO:0019221)	Chemokine activity (GO:0008009) Chemokine receptor binding (GO:0042379) Cytokine activity (GO:0005125)	Secretory granule membrane (GO:0030667) Azurophil granule (GO:0042582) Vacuolar lumen (GO:0005775)	Cytokine–cytokine receptor interaction (map04060) Chemokine signaling pathway (map04062) Viral protein interaction with cytokine and cytokine receptor (map04061)
GSE64610	Inflammatory response (GO:0006954) Cellular response to lipopolysaccharide (GO:0071222) Cytokine-mediated signaling pathway (GO:0019221)	Cytokine activity (GO:0005125) Chemokine receptor binding (GO:0042379) Cytokine activity (GO:0005125)	Microvillus membrane (GO:0031528) Golgi membrane (GO:0000139) Cytoskeleton (GO:0005856)	Rheumatoid arthritis (map05323) Viral protein interaction with cytokine and cytokine receptor (map04061) Toll-like receptor signaling pathway (map04620)
GSE63931	Neutrophil chemotaxis (GO:0030593) Granulocyte chemotaxis (GO:0071621) Neutrophil migration (GO:1990266)	Chemokine activity (GO:0008009) Chemokine receptor binding (GO:0042379) Serine-type endopeptidase activity (GO:0004252)	Cytolytic granule (GO:0044194) Lysosome (GO:0005764) T cell receptor complex (GO:0042101)	Viral protein interaction with cytokine and cytokine receptor (map04061) Toll-like receptor signaling pathway (map04620) Chemokine signaling pathway
GSE55664	Natural killer cell mediated immunity (GO:0002228) Chemokine-mediated signaling pathway (GO:0070098) Cellular response to chemokine (GO: 1990869)	Chemokine activity (GO:0008009) Chemokine receptor binding (GO:0042379) CCR chemokine receptor binding (GO: 0048020)	Cytolytic granule (GO:0044194) Ficolin-1 rich granule membrane (GO:0101003) Ficolin-1 rich granule (GO:0101002)	Viral protein interaction with cytokine and cytokine receptor (map04061) IL-17 signaling pathway (map04657) Staphylococcus aureus infection (map05150)
GSE42088	Positive regulation of acute inflammatory response (GO:0002675) negative regulation of transcription from RNA polymerase II in response to stress (GO:0097201) positive regulation of fever generation (GO:0031622)	Protein kinase activator activity (GO:0030295) cAMP response element binding (GO: 0035497) Kinase binding (GO:0019900)	Macropinosome (GO:0044354) Pinosome (GO:0044352) Endocytic vesicle membrane (GO: 0030666)	TNF signaling pathway (map04668) Rheumatoid arthritis (map05323) IL-17 signaling pathway (map04657)
GSE43661	Heat acclimation (GO:0010286) Cellular heat acclimation (GO:0070370) Regulation of neuroblast proliferation (GO:1902692)	Chemokine activity (GO:0008009) Chemokine receptor binding (GO:0042379) C3HC4-type RING finger domain binding (GO:0055131)	Aggresome (GO:0016235) Cornified envelope (GO:0001533) Multivesicular body, internal vesicule (GO:0097487)	Legionellosis (map05134) Splicesome (map03040) Antigen processing and presentation (map04612)

### Construction of PPI network and identification of central hub genes

**Figure 4:**
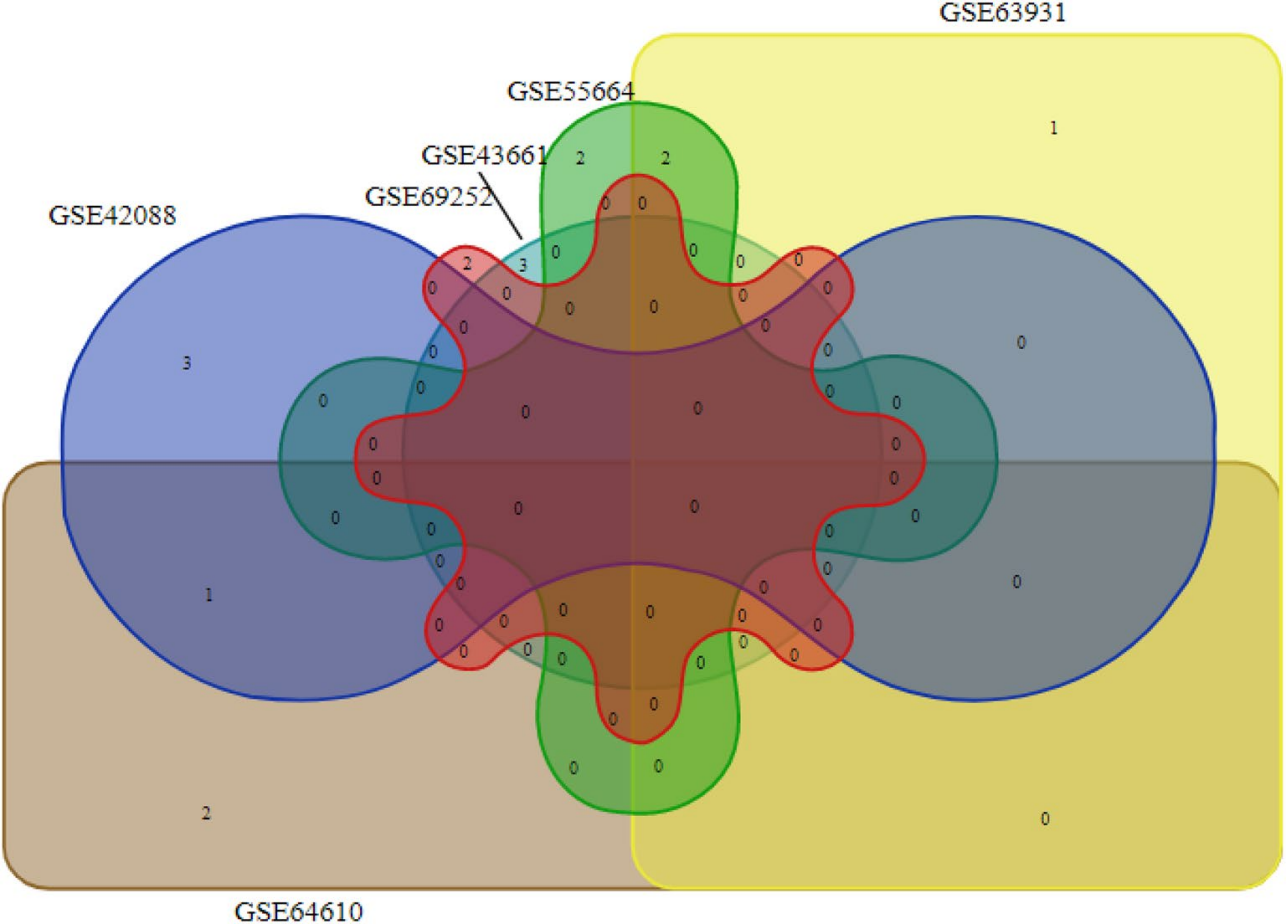
The overlap of hub genes between all analyzed datasets is shown by Venn diagram. Accordingly, there are no common genes between all of these datasets.

PPI networks serve as numerical depictions of the actual physical interactions that occur among proteins within the cell. The results of the number of nodes and edges of the examined datasets are listed in Table 4. The ultimate selection of hub genes was established by identifying the intersection between the sets of hub genes obtained from three algorithms (Betweenness, closeness, and degree) and the resultant of MCODE^25^. The findings indicated that *SOCS3, TNFAIP3, JUN* and, *TNF* were the hub genes identified for GSE42088. In addition, *CCR7* and *IDO1* were recognized as the hub genes that were identified for GSE69252. Our results shown that *CXCL8, CXCL9*, and *CXCL10* are hub genes in GSE55664. Additionally, *CXCL9*, *CXCL10*, and *FCGR3A* represent the hub genes identified in relation to GSE63931. Moreover, *VEGFA*, *IL1B* and *TNF* were found as hub genes for GSE64610. Finally, *LRRK2, HSPA1B*, *CXCL9,* and, *RPL13* recognized to be the hub genes for GSE43661 dataset (Fig. 4 and Table 5).

**Table 4:** The results of examining protein interaction and the number of nodes and edges after removing disconnecting nodes in the network.

Dataset	Number of nodes	Number of edges	Average node degree
GSE55664	370	410	2.01
GSE42088	94	112	1.92
GSE43661	172	191	2.1
GSE63931	65	69	1.35
GSE64610	104	114	2.19
GSE69252	61	71	2.02

**Table 5:** The sets of hub genes obtained from three algorithms (Betweenness, closeness, and degree).

Genes	Betweenness centrality	Closeness centrality	Degree
*VEGFA*	0.763514051	0.352549889	51
*RPL13*	0.301170289	0.206225681	27
*CXCL9*	0.435037019	0.263681592	27
*CCR7*	0.193323249	0.256865913	18
*CXCL10*	0.400551973	0.28342246	13
*SOCS3*	0.122203646	0.181299886	11
*TNFAIP3*	1	1	9
*TNF*	0.079638033	0.241274659	8
*JUN*	1	1	7
*FCGR3A*	1	1	6
*LRRK2*	0.037497015	0.175690608	4
*IDO1*	1	1	4
*CXCL8*	1	1	4
*IL1B*	0.025077621	0.21003963	3

### Validation of hub genes

**Figure 5:**
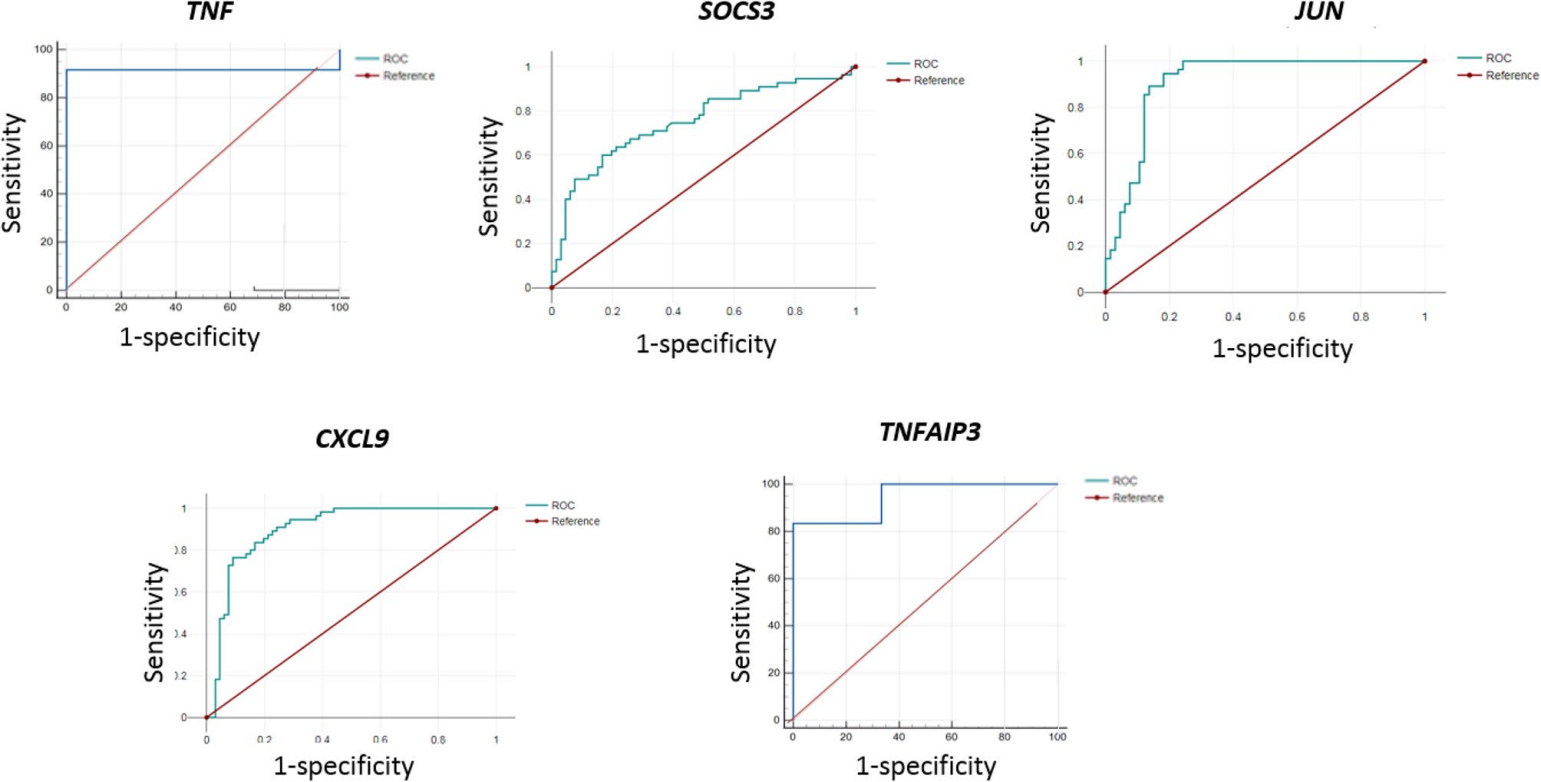
This figure showed the evaluation of sensitivity and specificity of hub genes in the diagnostic of the infection.

By utilizing the ROC curve (AUC > 0.6) to assess the prognostic value of hub genes in the PPI network^26^, 5 out of 14 hub genes, namely, *TNF, SOCS3, JUN*, *TNFAIP3*, and *CXCL9* showed potential indications as potential infection biomarkers (Table 6). However, the remaining genes, were not validated, probably owing to the limited number of samples. To ensure the accuracy and reliability of these genes as potential biomarkers, conducting further investigations using larger sample groups is highly recommended. The area under the ROC curve (AUC) reflects diagnostic value of the test (Fig. 5).

**Table 6:** Classification of hub genes based on *Leishmania* spp. and cell sources evaluated in datasets.

Hub genes	Species	Sample source
*TNF*	*L. major/L. donovani*	Human dendritic cells
*SOCS3*	*L. major*	Human dendritic cells
*JUN*	*L. major*	Human dendritic cells
*TNFAIP3*	*L. major*	Human dendritic cells
*CXCL9*	*L. major /L. braziliensis*	Human macrophage cells /skin biopsy

### Identification of miRNA-hub genes interaction

**Figure 6:**
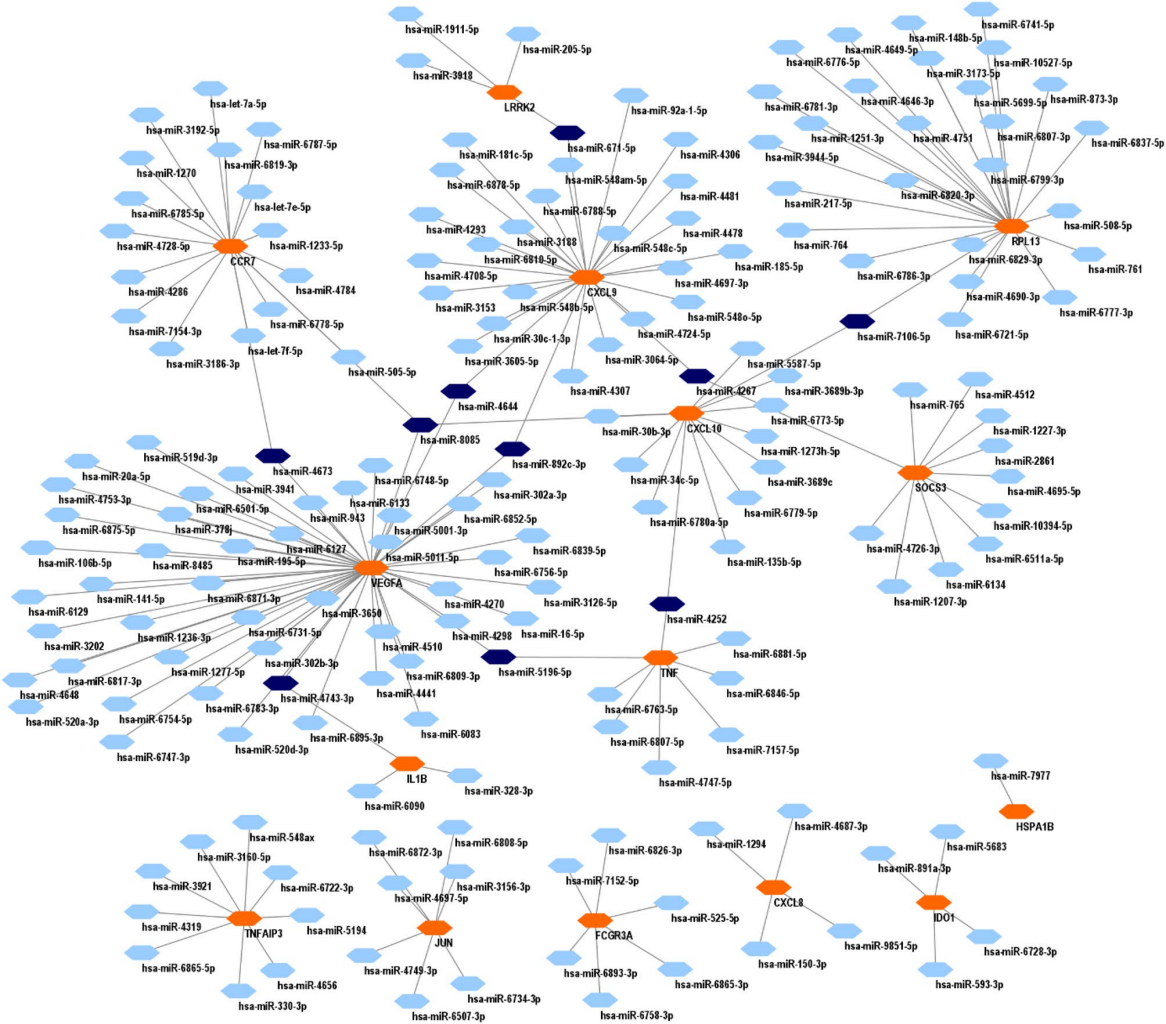
The interaction network between miRNAs and hub genes in miRWalk. Blue nodes represent miRNAs, while orang node displays hub genes.

Based on the result obtained from miRWalk, *hsa-miR-8085, hsa-miR-4673, hsa-miR-4743-3p, hsa-miR-892c-3p, hsa-miR-4644, hsa-miR-671-5p, hsa-miR-7106-5p, hsa-miR-4267, hsa-miR-5196-5p, hsa-miR-4252* display a substantial degree of interaction with the majority hub genes which candidate them as principal miRNA signatures of cell specific infections (Fig. 6 and Table 7).

**Table 7:** hub miRNAs and interaction of miRNA-hub genes based on statistically parameters.

miRNA	Degree	Targeted gene	Betweenness centrality	Closeness centrality
*hsa-miR-8085*	3	*CXCL10, VEGFA,* *CCR7,*	0.421224425	0.321212121
*hsa-miR-4673*	2	*VEGFA, CCR7*	0.066767508	0.276521739
*hsa-miR-4743-3p*	2	*IL1B, VEGFA*	0.03725818	0.263681592
*hsa-miR-892c-3p*	2	*CXCL9, VEGFA*	0.190948173	0.30170778
*hsa-miR-4644*	2	*CXCL9, VEGFA*	0.190948173	0.30170778
*hsa-miR-671-5p*	2	*LRRK2, CXCL9*	0.049359127	0.211155378
*hsa-miR-7106-5p*	2	*CXCL10, RPL13*	0.283735371	0.239097744
*hsa-miR-4267*	2	*CXCL9, SOCS3*	0.129607515	0.215155616
*hsa-miR-5196-5p*	2	*TNF, VEGFA*	0.062707322	0.268128162
*hsa-miR-4252*	2	*TNF, CXCL10*	0.026722925	0.226173542

In this study, in addition to evaluating miRNAs through gene-miRNAs interaction, we also conducted a comprehensive review of literature studies. The reviewed studies are presented in Table 8. Based on the comparison between the results of the present study and previous ones, some miRNA hubs play an important role during *Leishmania* spp. infection and probably during disease progression.

**Table 8:** Upregulated miRNAs found in literature reviews related to our key miRNA infection-associated networks in different clinical forms of leishmaniasis.

Predicted miRNA	Year	*Leishmania* species	References
miR-146a-3p and miR-146a-5p	2020	*L. major*	^27^
hsa-miR-146, miR-9, miR-106, and mir-324	2020	*L. donovani*	^28^
Up-regulation of miR-155	2021	*L. major*	^29^
let7i-5p, miR-30e-5p, miR-302a-3p, miR-302b-3p, miR-34c-5p	2021	*L. (V.) braziliensis* promastigote-infected THP-1 Cell	^30^
miR-302b-3p, miR-372-3p, miR-373-3p	2022	*L. infantum*	^31^

## Discussion and conclusion

Leishmaniasis, a parasitic illness caused by protozoan parasites, is commonly found in several regions of the tropics, subtropics, and southern Europe^32^. It is an infectious disease caused by *Leishmania* parasites and transmitted through the bite of phlebotomine sand flies, is classified as a neglected tropical disease (NTD) with a number of associated risk factors^33^. The diagnosis and treatment of this particular parasitic infection, which is caused by a variety of *Leishmania* parasite species, presents considerable challenges^34^. Consequently, the discovery of reliable biomarkers or molecular targets if including therapeutics offers enormous potential for improving disease management and patient outcomes^35^.

In this article, we explored the gene expression profile of individuals infected with various *Leishmania* spp. through analyzing multiple microarray datasets, in order to uncover potential biomarkers such as genes and miRNAs for *Leishmania* infections, highlighting their implications for diagnosis, treatment monitoring, and future research directions. Accordingly, the abnormal expression of miRNAs could serve as effective and non-invasive biomarkers for diagnosing and predicting various disorders, including infectious diseases^18^.

Since the raw data of GSE64610 dataset included the total data of both species, we reported the total number of genes. Although in our study the number of infected (97) and normal (54) samples were not in numerical balance, this issue could not affect the final statistical balance and we obtained acceptable data concerning DEG's. In addition, we rigorously filtered the obtained DEG's and determined a limited number of final hub genes. Accordingly, other studies have also recruited the same conditions. For example, in Li et al.’s study^36^, the number of infected samples (33) and normal (18) samples were not in numerical balance. Hence, the obtained DEGs (108 upregulated, 10 downregulated) are actually the sum of the DEGs in samples infected with *L. donovani* and *L. major*. Some of these genes have had expression changes in *L. donovani* and some in *L. major*.

Our study has identified the *TNF* gene as a potential hub gene in the context of differential *Leishmania* infections. This gene has a significant impact on the immune response during *Leishmania* infection, mainly by controlling the production and activation of pro-inflammatory cytokines, including *TNF-α*^37^*.* Evidences demonstrated a notable increase in *TNF-α* concentration in the serum of patients across all age groups with CL when compared to their respective control groups^38,39^. The function of TNF-α in Leishmania infections is a subject of debate, but many studies suggest that moderate amounts of TNF-α can aid in the clinical outcome of *Leishmania* infection by stimulating T-helper 1 (Th1) response^40^. Nevertheless, high levels of this cytokine can lead to tissue damage and the formation of persistent lesions. Despite the existence of numerous polymorphisms within the *TNF* gene locus, it is not yet certain whether these polymorphisms have a direct impact on TNF-α levels^41^. This suggests that the *TNF* gene might be linked to how susceptible people are to leishmaniasis. However, further research is needed to fully understand the role of the *TNF* gene in leishmaniasis^42^.

Additionally, *SOCS3* was another potential hub gene in this context. *SOCS3*, a gene known for regulating the immune responses in various infections, plays a crucial role in *L. major* infection. Mathematical modeling revealed that the critical role of the *SOCS1/SOCS3* ratio in bolstering the early immune response^43^. This ratio was quantified both computationally and experimentally, signified an essential immune axis that regulates macrophage phenotypes during *L. major* infection. Notably, *SOCS1*'s ability to inhibit the JAK/STAT1 signaling pathway, leading to the suppression of pro-inflammatory cytokine expression^44^, positions it as a promising candidate for therapeutic intervention in leishmaniasis. Studies have demonstrated that transgenic mice with increased levels of *SOCS3* gene expression exhibit heightened airway responsiveness, indicating that the expression of *SOCS3* in CD4^+^ T cells encourages Th2-dependent responses like allergic reactions^45^. Additionally, it has been discovered that excessive *SOCS3* expression in T cells also contributes to the advancement of leishmaniasis by promoting a dominant IL-4 response. Furthermore, abnormal IL-4 production in the initial stages of infection is responsible for the disease progression in transgenic *SOCS3* mice^46^.

*JUN* as another potential hub gene, is known to play a pivotal role in regulating immune responses to bacterial infections by modulating the expression of critical cytokines and chemokines^47^. While there is no direct evidence of *JUN* ‘s role in the immune response to leishmaniasis, its involvement in the regulation of the immune response to other infections suggests it might also affect how our body responds to leishmaniasis^48^. In addition, in our study, there is evidence to suggest that the vascular endothelial growth factor *(VEGF)* plays a role in the pathogenesis of *Leishmania* infection specially during lymphangiogenesis, or the formation of new blood vessels. It is also involved in the regulation of immune responses and has been implicated in the development and progression of several diseases, including cancer and infectious diseases^49^. Several studies have investigated the relationship between *VEGF* gene and *Leishmania* infection^50^. Tiffani et al. have shown that macrophages are the predominant cell type expressing VEGF-A during *L. major* infection. Given that *Leishmania* parasites activate hypoxia-inducible factor 1α (HIF-1α) and this transcription factor can drive VEGF-A expression^51^. These findings also suggest that targeting *VEGF* signaling may be a potential therapeutic strategy for the treatment of leishmaniasis^52^.

The immune response to *Leishmania* infection is complex and involves a variety of immune cells and biomolecules. One important differentially activated immune gene in this response is CXCL9, a chemokine codifying gene that is expressed by infected macrophages leading to the recruitment of T cells to the site of infection and inflammation been thus implicated in a number of disease conditions like in severe diseases and also in favoring M1 macrophage polarization upon infections^53^. In laboratory studies on visceral leishmaniasis, infected kupffer cells quickly release CCL2, CCL3, and CXCL10. These molecules attract inflammatory monocytes and T cells, leading to the formation of a granuloma^54^. There is a direct relationship between the severity of splenic damage and the levels of *CXCL9, CXCL10, IFN-γ,* and *IL-10* in the blood during VL. This suggests that the immune system responds to high levels of parasitic activity by producing both pro-inflammatory and regulatory molecules to control parasitemia. The host may use this strategy to limit parasite growth during VL^55^. This process plays a crucial role in initiating an immune response against the parasites. Several studies have investigated the role of *CXCL9* in leishmaniasis. In a study by Gomes et al., *CXCL9* levels were found to be elevated in individuals with cutaneous leishmaniasis, suggesting that it may be a useful biomarker for monitoring disease progression and treatment efficacy^56^. Similarly, a study by de Brito et al. found that *CXCL9* levels were elevated in individuals with cutaneous leishmaniasis, and that treatment with pentoxifylline drugs led to a decrease in *CXCL9* levels^57^. Overall, the literature suggests that *CXCL9* plays an important role in the immune response against leishmaniasis, and may be a useful biomarker for monitoring disease progression and treatment efficacy^58^. In addition, targeting this molecule for macrophage polarization modulation might be a possible therapeutic strategy.

*TNFAIP3*, also known as *A20*, was identified as a gene whose expression is rapidly induced by the Tumor Necrosis Factor (TNF). This gene encodes a ubiquitin-editing enzyme, which inhibit NF-kappa B activation as well as TNF-mediated apoptosis. TNFAIP3 protein is involved in the cytokine-mediated immune and inflammatory responses^59,60^. According to a previous investigation, *Leishmania* species inhibit the expression of *NLRP3* inflammasome, which is a multiprotein signaling platform. This, in turn, inhibits the activation of caspase-1 and the maturation of IL-1β. The reduction of NLRP3 and pro-IL-1β during infection is due to a decrease in NF-κB activity. This decrease in NF-κB activity is linked to an increase in the expression of TNFAIP1, which is a negative regulator of NF-κB signaling^61^. In another study, increased transcript abundance was observed in *L. amazonensis* infection for *TNFAIP3*^62^. Overall, RG1 was highly upregulated in VL (*L. donovani*) cases, and suppressed TLR-triggered proinflammatory responses through elevated reactive oxygen species stimulating *TNFAIP3*^63^. In line with these studies, our study also showed a significant increase in the expression of the *TNFAIP3* gene in patients compared to the control groups.

miRNAs are a type of non-coding RNA that have a vital role in regulating various cellular functions^64^. Their modulation and abnormal expression have been associated with the development of pathogenesis processes of several disorders, including infectious diseases such as leishmaniasis. Therefore, gaining a thorough understanding of how miRNAs interact with their targets could provide valuable insights into the underlying mechanisms of these diseases^15^.

Some research demonstrated a notable upregulation of miR-302b-3p, miR-372-3p, miR-373-3p, and miR-607 in THP-1 cells in response to intracellular parasitism compared to the healthy controls^31^. Additionally, differential miRNA expression profiles were observed in *L. (V.) braziliensis* promastigote-infected THP-1 cells, including let7i-5p, miR-30e-5p, miR-302a-3p, miR-302b-3p, and miR-34c-5p^30^. Furthermore, based on some studies, the upregulation of hsa-miR-146, miR-106, miR-324, miR-221, miR-9, and miR-155 in infected patients have the potential to downregulate the IFN-γ signaling pathway, contributing to disease progression during Post-Kala-Azar Dermal Leishmaniasis (PKDL)^65^. Our meta-analysis employing accessible datasets was able to clarify and identify putative biomarkers related to infection. As a result, the identification of five prospective gene biomarkers, such as *TNF, SOCS3, JUN*, *TNFAIP3*, and *CXCL9*, that are differently expressed in *Leishmania* infected cells, offers possibilities as clinically diagnostic biomarkers for an accurate identification of *Leishmania* infected individuals. Furthermore, protein interaction results revealed dysregulated biochemical pathways (cellular response to immunologic stimulus and response to inflammatory stimulus) that might be useful as therapeutic targets. The validation of these biomarkers and metabolic pathways by experimental and functional researches might provide future interesting prospects for the management of leishmaniasis.

We evaluated gene enrichment analysis to find the relevant pathways associated with *Leishmania* infection. Leishmaniasis is recognized for triggering an intricate immune reaction that encompasses both innate and adaptive immune responses. We have discovered that DEGs are enriched in pathways connected to the regulation of immune response, such as signaling pathways for cytokines (such as TNF, IL-12, IFN-gamma), NF-kappa B signaling, toll-like receptors and IL-17 signaling pathways. Hints of the immune system having an important function in fighting against leishmaniasis infection^66^. IL-17 is a cytokine with proinflammatory properties, primarily secreted by activated T cells, specifically CD4^+^ cells over CD8^+^ cells. This cytokine has been implicated in various inflammatory human diseases, such as rheumatoid arthritis and psoriasis^67^. In addition to T cells, IL-17 also stimulates other cell types, such as macrophages, to generate inflammatory mediators like TNFα, IL-1, and chemokines. These events ultimately result in the recruitment of neutrophils^68^. Previous studies have demonstrated that the increased recruitment of neutrophils, dependent on IL-17, into lesions of BALB/c mice infected with *L. major*, significantly contributes to the outcome of the disease^69^. Also increased levels of IL-17A in BALB/c mice infected with *L. major*, were linked to higher production of IL-23 by infected DC^70^.

Furthermore, we found that DEGs significantly enriched in pathways such as neutrophil/ granulocyte chemotaxis and migration. Neutrophils serve as the initial defense against infection and are promptly summoned to the infection site once the parasite infiltrates the host. While they possess the capability to engulf the *Leishmania* parasites, their capacity to eliminate the parasites remains limited. Besides, neutrophils release antimicrobial peptides and reactive oxygen species to aid in the management of the infection. Neutrophil and macrophages both utilize autophagy to degrade and recycle cellular component, including pathogens^71^. Furthermore, the enrichment of cellular components such as “Azurophil granule”, “Lysosome”, and “Cytolytic granule” may indicate an enhanced neutrophil response to the infection, highlighting the importance of these cells in the immune defense against *Leishmania*.

In conclusion, our meta-analysis employing accessible datasets was able to clarify and identify putative biomarkers related to cell specific *Leishmania* infections. As a result, since key immune responses can be considered as potential correlates of immunity against infectious pathogens thus having potential diagnostic value, the identification of five prospective immune response genes that are differently expressed in the infected cells, such as *TNF, SOCS3, JUN*, *TNFAIP3*, and *CXCL9*, offers possibilities to serve as biomarkers to identify *Leishmania* infected individuals. Furthermore, protein interaction research revealed major pathways linked to probable biological events such as various dysregulated biochemical pathways (cellular response to immunologic stimulus and response to inflammatory stimulus). Whit the aim to select key genes and miRNAs regulating the pathogen-host interactions and thus potentially useful for disease management particularly at the early stages of inflammatory responses, we have here approached in silico tools that predict the biological roles of human genes and miRNAs. Of course, all these predictions have to be well validated as in many (it not almost all) initial in silico studies. A thorough functional driven investigation of these biomarkers with well controlled samples including different cells and *Leishmania* spp. will provide new interesting research papers to improve disease management. As a summary, it seems that we need to go deeper, particularly in the study of microRNAs. The validation of these biomarkers and metabolic pathways by experimental and functional research might fully confirm the current study’s findings.

## Data Availability

The authors confirm that the data supporting the findings of this study is available within the article.
